# The effect of inspiratory muscle training and detraining on the respiratory metaboreflex

**DOI:** 10.1113/EP090779

**Published:** 2023-02-08

**Authors:** Jason S. Chan, Leah M. Mann, Connor J. Doherty, Sarah A. Angus, Benjamin P. Thompson, Michaela C. Devries, Richard L. Hughson, Paolo B. Dominelli

**Affiliations:** ^1^ Department of Kinesiology and Health Sciences Faculty of Health University of Waterloo Waterloo Ontario Canada; ^2^ Schlegel‐UW Research Institute for Aging Waterloo Ontario Canada

**Keywords:** arterial blood pressure, heart rate, respiratory muscles

## Abstract

Respiratory muscle training (RMT) improves respiratory muscle (RM) strength and attenuates the RM metaboreflex. However, the time course of muscle function loss after the absence of training or ‘detraining’ is less known and some evidence suggest the respiratory muscles atrophy faster than other muscles. We sought to determine the RM metaboreflex in response to 5 weeks of RMT and 5 weeks of detraining. An experimental group (2F, 6M; 26 ± 4years) completed 5 weeks of RMT and tibialis anterior (TA) training (each 5 days/week at 50% of maximal inspiratory pressure (MIP) and 50% maximal isometric force, respectively) followed by 5 weeks of no training (detraining) while a control group (1F, 7M; 24 ± 1years) underwent no intervention. Prior to training (PRE), post‐training (POST) and post‐detraining (DETR), all participants underwent a loaded breathing task (LBT) to failure (60% MIP) while heart rate and mean arterial blood pressure (MAP) were measured. Five weeks of training increased RM (18 ± 9%, *P* < 0.001) and TA (+34 ± 19%, *P* < 0.001) strength and both remained elevated after 5 weeks of detraining (MIP‐POST vs. MIP‐DETR: 154 ± 31 vs. 153 ± 28 cmH2O, respectively, *P* = 0.853; TA‐POST vs. TA‐DETR: 86 ± 19 vs. 85 ± 16 N, respectively, *P* = 0.982). However, the rise in MAP during LBT was attenuated POST (−11 ± 17%, *P* = 0.003) and DETR (−9 ± 9%, *P* = 0.007) during the iso‐time LBT. The control group had no change in MIP (*P* = 0.33), TA strength (*P* = 0.385), or iso‐time MAP (*P* = 0.867) during LBT across all time points. In conclusion, RM and TA have similar temporal strength gains and the attenuation of the respiratory muscle metaboreflex remains after 5 weeks of detraining.

## Abstract (highlights)

- **What is the central question of this study?**
  Is the attenuation of the respiratory muscle metaboreflex preserved after detraining?
- **What is the main finding and its importance?**
  Inspiratory muscle training increased respiratory muscle strength and attenuated the respiratory muscle metaboreflex as evident by lower heart rate and blood pressure. After 5 weeks of no inspiratory muscle training (detraining), respiratory muscle strength was still elevated and the metaboreflex was still attenuated. The benefits of inspiratory muscle training persist after cessation of training, and attenuation of the respiratory metaboreflex follows changes in respiratory muscle strength.

## 1 INTRODUCTION

As the primary muscle for respiration, the diaphragm has certain characteristics to manage a wide range of ventilatory demands. For example, the diaphragm is a skeletal muscle composed of approximately 50% slow twitch (type I) and 50% fast twitch (type II) muscle fibres (Mizuno & Secher, 1989). This balance of muscle fibres in the diaphragm allows for the maintenance of normal alveolar ventilation (which requires the diaphragm to be continuously active) as well as brief periods of higher force outputs. The diaphragm can be difficult to compare to other skeletal muscles due to the unique properties of the diaphragm muscle. A potential comparable skeletal muscle is the tibialis anterior (TA), which has similar composition of muscle fibres (∼40% type I and 60% type II) (Yang & Yoo, 1986) resulting in similar fatigue resistance and energetics. In addition, in healthy mobile individuals, the TA is active throughout the day as it lifts the foot during locomotion. Furthermore, a relatively low percentage of total TA strength is used during walking (Gomes et al., 2017). The TA is also not likely to be receiving additional specific training unlike other skeletal muscles, such as the quadriceps or biceps, which are often part of regular exercise regimes. The lack of specific training will minimize the effects of muscle memory, as muscle memory allows for a faster gain in muscle mass to a muscle that has been previously trained (Gundersen, 2016). As a result, the strength response of the TA is a reasonable comparison to the strength response of the diaphragm as these muscles are akin.

During spontaneous breathing, the diaphragm only fatigues following prolonged high intensity exercises, indicating high force generation (Babcock et al., 1995) and likely changes in blood chemistry (i.e. pH, lactic acid, etc.) activating the respiratory muscle metaboreflex (Dempsey et al., 2006). Functionally, the respiratory muscle metaboreflex operates and achieves the same goals as other metaboreflexes (Fisher et al., 2015), but there is evidence suggesting the respiratory muscles may be prioritized. For example, increasing the work of breathing has been shown to reduce blood flow in working locomotor muscles during exercise and increase respiratory muscle blood flow via sympathetic modulation (Dominelli et al., 2017, 2019; Harms et al., 1997; Sheel et al., 2018). A potential mechanism to explain prioritized blood flow to the respiratory muscles has been discovered in rats, whereby the arterioles in the diaphragm are less sensitive to α‐adrenergic constriction compared to the arterioles in the gastrocnemius (Aaker & Laughlin, 2002).

Inspiratory muscle training (IMT) is effective in increasing the strength of the respiratory muscles (Ramsook et al., 2017; Romer & McConnell, 2003; Witt et al., 2007) and that as little as 5 weeks of training is sufficient to blunt the respiratory muscle metaboreflex response (Witt et al., 2007). For instance, 5–9 weeks of IMT could attenuate the competition of blood flow between respiratory muscles and other working limb muscles during strenuous exercise (Ramsook et al., 2017; Romer & McConnell, 2003; Romer et al., 2006; Witt et al., 2007). There is also evidence that respiratory muscle training reduced fatigue in other exercising locomotor muscles via improving vascular conductance and blood flow in these muscles in chronic heart failure patients (Chiappa et al., 2008). Currently, only one study has assessed the effect of complete detraining (defined as the cessation of any respiratory muscle training) on respiratory muscle function and found that 9 weeks of detraining (after 9 weeks of training), decreased MIP by 7% from peak levels; however, there were no further significant decreases over another 9 weeks of detraining (18 weeks total) (Romer & McConnell, 2003). The same study found that in a separate group of participants, a reduction of the training frequency by 66% was sufficient to maintain the increased inspiratory muscle function up to 18 weeks (Romer & McConnell, 2003). However, the response of the metaboreflex to detraining remains unknown. It is important to understand any changes in the respiratory metaboreflex in the absence of training because it is unlikely that individuals would engage in IMT indefinitely. For example, IMT may be recommended for those who enrol in structured pulmonary rehabilitation (Spruit et al., 2013) and such programmes are often repeated (Heng et al., 2014). By determining, as a first step, how a healthy individual's respiratory metaboreflex responds to detraining, others can follow up to determine optimal time between training bouts.

Accordingly, the purpose of this study was the following. First, to evaluate the respiratory muscles and TA in terms of strength changes over the course of 5 weeks of training and detraining, the latter being defined as the absence of IMT. We sought to utilize 5 weeks of training and detraining in order to (i) verify previous work that investigated the metaboreflex after 5 weeks of IMT and (ii) have similar training and detraining times. We hypothesized that the strength response to training in both muscles would be preserved after detraining. Secondly, our primary purpose was to determine if the attenuation of the respiratory muscle metaboreflex after IMT would persist after 5 weeks of detraining. We hypothesized that the attenuation of the respiratory muscle metaboreflex would persist after 5 weeks without IMT (detraining) due to the elevated strength compared to baseline.

## 2 METHODS

### 2.1 Ethical approval

The study was approved by the Clinical Research Ethics Board at the University of Waterloo (Approval number: 41928) and adheres to the *Declaration of Helsinki*, except for registration in a database. All participants were informed of the experimental procedures and potential risks involved, and provided written, informed consent.

### 2.2 Participants

Sixteen young healthy participants were recruited for this study and were randomly assigned into either the control (*n* = 7 male, *n* = 1 female) or experimental (*n* = 6 male, *n* = 2 female) group after ensuring balanced sexes in each group. Participants with symptoms of cardiovascular, metabolic or respiratory disease, a history of smoking, and/or taking any prescription medicine that may influence their response to exercise were excluded. Only females on monophasic birth control were included to minimize the known effect of the menstrual cycle on the metaboreflex (Parmar et al., 2018). Females were always tested during the active phase of the monophasic birth control to minimize hormone‐related variation. All participants engaged in physical activity >3 days/week, but none were engaging in intensive aerobic or strength training. All participants were instructed to keep activity levels consistent throughout the protocol and to report any major changes (e.g., injury or illness); none reported any.

### 2.3 Experimental overview

All participants completed a 10‐week protocol with three identical laboratory testing sessions termed pre‐training (PRE), post‐training (POST) and post‐detraining (DETR). Each laboratory session consisted of measurements of maximal respiratory muscles and dorsiflexor strength along with an inspiratory threshold loading protocol designed to elicit the respiratory metaboreflex. The experimental group also completed a 5‐week inspiratory muscle and dorsiflexor training protocol (weeks 1–5) followed by a 5‐week detraining protocol (no training; weeks 6–10). The control group did not perform any training (respiratory muscles or dorsiflexors) over the 10 weeks.

### 2.4 Maximal inspiratory, expiratory and dorsiflexor strength

Maximal inspiratory pressure (MIP) and maximal expiratory pressure (MEP) procedures were performed according to American Thoracic Society standards (Green et al., 2002). Briefly, to perform the MIP and MEP manoeuvres, participants were in an upright seated position with nose clips worn. The MIP manoeuvre was performed at the end of normal expiration (i.e., functional residual capacity) and the participant immediately inserted the mouthpiece with a pinhole opening and inhaled as hard as possible with verbal encouragement. The MEP manoeuvre was performed by having participants inhale to total lung capacity, immediately insert the mouthpiece and exhale as hard as possible with verbal encouragement. For both MIPs and MEPs, mouth pressure was measured via a calibrated differential pressure transducer (DP15‐32; Validyne Engineering, Northridge, CA, USA) connected to a port in the mouthpiece. In between each manoeuvre, participants were given 30 s to 1 min of rest. The average of three consistent (<5% difference) MIPs/MEPs was taken and used as the average for that experimental session.

Maximal isometric strength of dorsiflexors (one repetition maximum; 1RM) was measured with a shin isolator (Sky King, Oseola, IN, USA) attached to a load cell (SSM, Interface MFG, Scottsdale, AZ, USA) that was anchored to a metal platform. The participant was in the seated position and a strap secured the participant's leg to the chair, minimizing the recruitment of other muscles. The knee and ankle were kept at a 90^o^ and 340^o^ (i.e., 20^o^ down) angle, respectively, while performing the dorsiflexion. Prior to each manoeuvre, the participant was reminded to not move the leg and only hinge at the ankle joint. The average of three consistent (<5% difference) manoeuvres was used as the maximal isometric dorsiflexor strength for that session.

### 2.5 Respiratory muscle metaboreflex

A custom inspiratory threshold‐loading device was used for the loaded breathing task, which was similar to previous studies (Dominelli et al., 2018). The inspiratory threshold‐loading device had three airflow openings (inspiration, expiration and participant). The inspiratory end was temporarily sealed off via a plunger attached with weights. To generate inspiratory airflow, the participant had to generate sufficient pressure to overcome the gravitational pull of the weights (threshold load) (Dominelli et al., 2018). The expiratory side of the loader had a one‐way valve capable of remaining sealed during inspiratory efforts. Throughout all testing, participants were in an upright and seated position. After instrumentation, 10 min of eupnoeic and unloaded breathing (no weights) was collected to ensure a steady state baseline. Thereafter, participants began the loaded breathing task consisting of inspiring against a threshold load that required 60% of their MIP to generate airflow, at a breathing frequency of 15 breaths per minute (duty cycle of 50:50) throughout the loaded breathing task (Witt et al., 2007). During the loaded breathing task, carbon dioxide (CO_2_) was titrated to the inspiratory side to ensure participants remained isocapnic throughout. A guideline was provided on a computer monitor to provide real‐time visual feedback of mouth pressure to guide the necessary pressure needed to be generated. An audio cue was used to signal the participant to inspire or expire to ensure a consistent breathing frequency and duty cycle. Participants continued the loaded breathing task until voluntary exhaustion defined as three consecutive breaths failing to reach the target guideline.

### 2.6 Physiological measurements during respiratory metaboreflex test

Throughout the baseline, loaded breathing task and recovery, the following variables were collected. Expired flow was measured using a calibrated pneumotachometer (model 3813; Hans Rudolph, Shawnee, KS, USA) and breathing frequency, expired volume and minute ventilation were subsequently derived. The pneumotachometer was placed distal to the one‐way expiratory valve to ensure it was not exposed to the substantial negative pressure which can impact accuracy. A calibrated gas analyser (CD‐3Am; Applied Electrochemistry, Bastrop, TX, USA) was connected to a port distal to the one‐way valve to measure expired carbon dioxide and estimated end‐tidal CO2 concentrations. An electrocardiogram (ECG) (ISO‐4 Isolation Preamplifier, CWE inc, Ardmore, PA, USA) in the three‐lead configuration, was used to determine heart rate. Photoelectric plethysmography (Human NIBP Nano Interface, ADInstruments, Colorado Springs, CO, USA) was used to non‐invasively measure arterial blood pressure via a cuff placed on the middle phalanx of the middle or ring finger on their warmed right hand to collect beat‐by‐beat blood pressure. An appropriately sized cuff was used for each participant and was consistent for each testing day. Throughout data collection, the participants’ right hand lay flat on a table, and they were asked to ensure it remained relaxed. All blood pressures reported were corrected to the level of the aorta using a height correction sensor fixed over the heart. An automated blood pressure cuff was used during rest to verify the absolute values for the beat‐by‐beat blood pressure.

### 2.7 Strength training protocol

The training performed by the experimental group occurred 5 days a week at home and consisted of two separate exercises. The first exercise consisted of two sets of 30 inspirations at 50% of their MIP using a commercially available respiratory muscle trainer (PBK3; PowerBreathe, Southam, UK). The other exercise included two sets of 30 repetitions of dorsiflexion at 50% of maximal isometric strength of the dorsiflexors of the dominant leg using the same device as described above (Shin Isolator). Weekly baseline measurement for MIP and 1RM of the dorsiflexors were taken to adjust the training for the week to maintain training progression (i.e., ensuring 50% of MIP/1RM). The experimental group also came to the laboratory weekly during detraining. Participants received daily reminders to verify all training had occurred. Adherence was also verified via the training log on the respiratory muscle trainer. All participants completed the entire training protocol and adherence for each session was >95% for every participant. The 5‐week detraining protocol started immediately after the 5 weeks of training and involved the complete cessation of inspiratory and dorsiflexor specific training. Throughout, participants were asked to keep any other exercise routines consistent. The participants in the control group did not perform any respiratory muscle or dorsiflexor training.

### 2.8 Data analysis

The values for MIPs and MEPs were calculated from the 1 s average of a nadir and peak pressures, respectively, which is consistent with international standards (Green et al., 2002). The mean value of maximal isometric strength of the dorsiflexors was calculated from the highest force generated over 1 s. Several time points were used during the metaboreflex trial for comparison between the three testing days: last minute of baseline; minutes 1, 2, and 3; the final minute and iso‐time of the threshold breathing task. The first 3 min of threshold loading provided the time‐dependent progression of the physiological responses. Since the duration of the metaboreflex trial was expected to increase with training, we compared both the last minute of each test and an iso‐time defined as the final minute from the pre‐training trial. Thus, the iso‐time allowed for comparison of the cardiovascular response to the loaded breathing task where equal amounts of respiratory muscle work had been completed during the PRE, POST and DETR trials.

Respiratory work was calculated via the integral of the mouth pressure generated for each breath and the following equation to determine the overall amount of work done:

(1) $$W_{\mathrm{tot}}=\left(\int P_{\mathrm{mouth}}\right)\left(F_{b}\right)\left(T_{\mathrm{exhaustion}}\right).$$

### 2.9 Statistical analysis

Similar to others who investigated the respiratory muscle metaboreflex after training, we examined the changes in each group independently (Witt et al., 2007). Specifically, the change in mean arterial pressure (MAP) and heart rate (HR) at iso‐time from rest of the loaded breathing task, the peak MIP, MEP and maximal isometric strength of the TA were compared with a one‐way repeated measures ANOVA. The weekly changes in respiratory or dorsiflexors muscle strength was compared with a one‐way repeated measures ANOVA. When significant *F*‐ratios were detected a Tukey *post hoc* test was used to determine where the differences lay. For determining the weekly changes, we compared each week to the PRE for the training arm or POST for the detraining arm. An independent Student's *t*‐test was used to compare the participant demographics between groups. Pearson product moment correlations were performed to determine the following relationships: inspiratory muscle strength (MIP) and the respiratory metaboreflex (HR and MAP); the change in MAP and change in time to exhaustion; and the starting strength (MIP and TA) and the change in strength (MIP and TA). All data are presented as means ± standard deviation and significance was set at *P* < 0.05.

## 3 RESULTS

### 3.1 Participants

There were no significant differences in age (26 ± 4 vs. 24 ± 1 years), height (176 ± 6 vs. 173 ± 8 cm), weight (76 ± 12 vs. 73 ± 16 kg), body mass index (25 ± 3 vs. 25 ± 6 kg/m^2^), resting heart rate (76 ± 7 vs. 81 ± 13 bpm) and resting mean arterial pressure (94 ± 8 vs. 92 ± 10 mmHg) for experimental and control groups respectively (all *P* > 0.05). Resting heart rate did not differ between the laboratory testing sessions for either group (EXP: 76 ± 7, 80 ± 9, 83 ± 11 bpm, *P* = 0.31, vs. CONT: 81 ± 13, 79 ± 9, 79 ± 12 bpm, *P* = 0.16, for PRE, POST and DETR, respectively). Similarly, resting mean arterial pressure was not different across testing sessions in the experimental or control group (EXP: 92 ± 7 vs. 95 ± 11 vs. 89 ± 7 mmHg, *P* = 0.08 vs. CONT: 92 ± 10 vs. 90 ± 10 vs. 86 ± 9 mmHg, *P* = 0.98 for PRE, POST and DETR, respectively). Based on predicted MIP (Hamilton et al., 1995), the experimental group and control group were not different in terms of percentage predicted MIP (118 ± 11% vs. 99 ± 22% for experimental and control groups, respectively).

### 3.2 Respiratory muscle and dorsiflexor strength response

**FIGURE 1:**
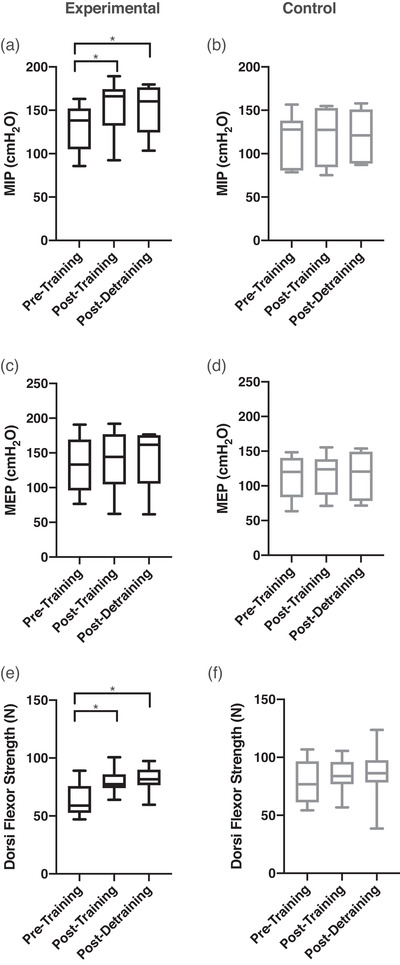
Box and whisker plot for maximal inspiratory pressure (MIP), maximal expiratory pressure (MEP) and tibialis anterior strength on each testing day for both groups. (a, b) MIP at pre‐training, post‐training and post‐detraining for the experimental (black, a) and control (grey, b) group. (c, d) MEP at pre‐training, post‐training and post‐detraining for the experimental (black, c) and control (grey, d) group. (e, f) TA strength at pre‐training, post‐training and post‐detraining in the experimental (black, e) and control (grey, f) group. *Significant differences (*P* < 0.05) from pre‐training values.

The change in respiratory muscle and dorsiflexor strength over the course of the study is shown in Figure 1 and Table 1. Compared to PRE, the experimental group had a significant increase in MIP at POST (+18 ± 9%, *P* < 0.001) that was minimally attenuated at DETR (+17 ± 7% relative to PRE, *P* < 0.001). The control group showed no significant difference in MIP across all time points (*P* = 0.33). There was a significant increase in MIP by week 2 and by week 3 for the dorsiflexors (Table 1). For reasons unrelated to the study (COVID‐19 restrictions) we were unable to collect week 7–9 in two participants for the detraining arm, and as such the detraining data for Table 1 are presented at *n* = 6. There were no significant changes in respiratory muscle or dorsiflexors strength on a weekly basis. There was no relationship between starting MIP and the percentage increase in MIP after training (*r* ^2^ = 0.04, *P* = 0.60). There were no changes in MEP for either group at any time point (Figure 1c, d). Compared to PRE, dorsiflexor maximal force was significantly greater in the experimental group at POST (+34 ± 19%, *P* < 0.001) and DETR (+33 ± 18%, *P* < 0.001). There were no changes in dorsiflexor maximal strength in the control group at any time point (*P* = 0.385). There was no relationship between starting dorsiflexor strength and the percentage increase in strength after training (*r* ^2^ = 0.003, *P* = 0.89) in the experimental group.

**TABLE 1:** Weekly changes in respiratory and dorsiflexors muscle strength for the experimental group.

Weekly training response (*n* = 8)				
	MIP (cmH_2_O)	MIP (% Pre)	DF strength (N)	DF strength (% Pre)
Pre	131 ± 27	—	65 ± 14	—
Wk 1	138 ± 29	6 ± 8	72 ± 13	14 ± 22
Wk 2	146 ± 34^†^	12 ± 9	74 ± 14	16 ± 17
Wk 3	150 ± 33*	14 ± 11	79 ± 18^‡^	22 ± 22
Wk 4	152 ± 35*	16 ± 10	78 ± 15^‡^	22 ± 17
Wk 5	154 ± 32*	18 ± 8	86 ± 20^†^	34 ± 19
*P*	<0.0001		<0.0001	

Weekly detraining response (*n* = 6)				
	MIP (cmH_2_O)	MIP (% Post)	DF strength (N)	DF strength (% Post)
Post	150 ± 36	—	80 ± 12	—
Wk 6	148 ± 31	−1 ± 5	80 ± 12	0 ± 3
Wk 7	146 ± 32	−2 ± 7	76 ± 11	−5 ± 8
Wk 8	143 ± 30	−4 ± 10	74 ± 14	−7 ± 15
Wk 9	143 ± 33	−4 ± 11	79 ± 10	0 ± 9
Wk 10	148 ± 32	−1 ± 8	79 ± 10	−1 ± 12
*P* value	0.347		0.393	

* Significantly different from Pre (*P* < 0.0001).

^†^ Significantly different from Pre (*P* < 0.01).

^‡^ Significantly different from Pre (*P* < 0.05).

Abbreviations: DF, dorsiflexor; MIP, maximal inspiratory pressure; PRE, pre‐training; Wk, week.

### 3.3 Respiratory muscle metaboreflex

**FIGURE 2:**
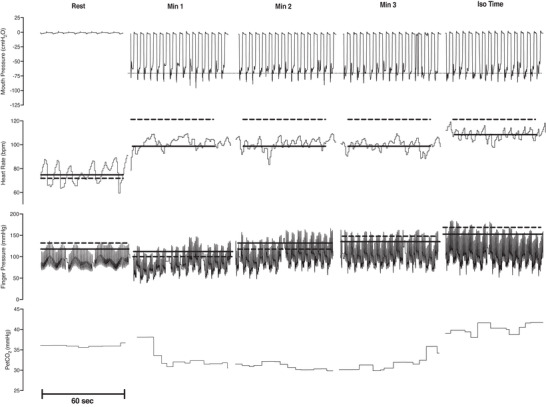
Raw data from a male participant in the experimental group at various time points during a loaded breathing task taken on the post detraining day. Loaded breathing was performed at 60% maximal inspiratory pressure with a 0.5 duty cycle at 15 breaths per minutes. Dotted line indicates mean value of the 1 min bin from pre‐training and continuous line indicates mean values from the post‐training. $P_{\mathrm{ETC}O_{2}}$, end‐tidal carbon dioxide pressure.

**FIGURE 3:**
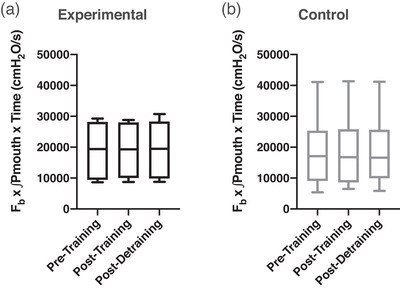
Box and whisker plot for respiratory work (*F*_b_ × ∫*P*_mouth_ × time; cmH_2_O/s) completed during the loaded breathing task on test days for both groups. The respiratory work completed at iso‐time at pre‐training, post‐training and post‐detraining in the experimental (a, black) and control (b, grey) group. *F*_b_, breathing frequency.

**FIGURE 4:**
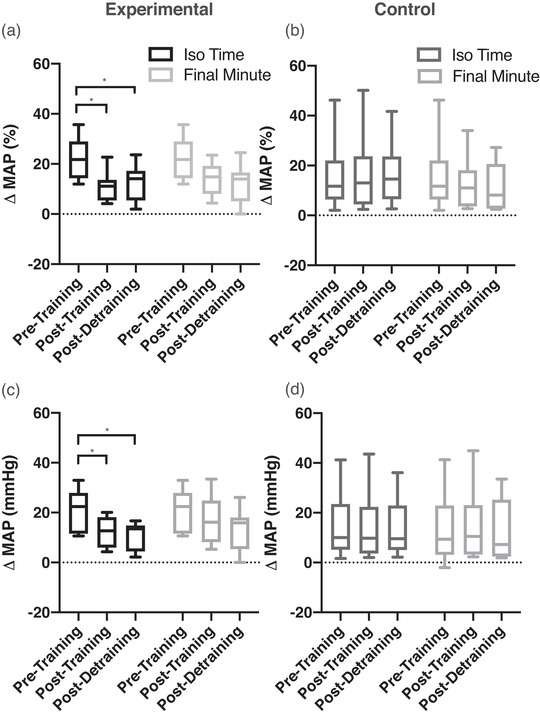
Box and whisker plot for percentage change of mean arterial blood pressure (MAP) and absolute change of MAP from rest during the loaded breathing task on each testing day for both groups. (a, b) Percentage change of MAP from rest to iso‐time of the experimental (black, a) and control (dark grey, b) group and rest to the final minute (light grey) of the loaded breathing task at pre‐training, post‐training and post‐detraining. (c, d) change of absolute MAP from rest to iso‐time of the experimental (black, c) and control (dark grey, d) group and rest to the final minute (light grey) of the loaded breathing task at pre‐training, post‐training, and post‐detraining. *Significant differences (*P* < 0.05) from pre‐training.

**FIGURE 5:**
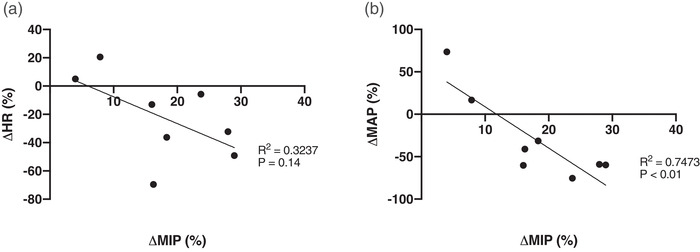
Relationship between the change in cardiovascular variables during the loaded breathing task from pre‐training to post‐training. (a) No relationship between the percentage change in heart rate and the change in maximal inspiratory pressure of the experimental group from pre‐training to post‐training (*P* = 0.14). (b) Strong relationship between the percentage change of mean arterial blood pressure and the change in maximal inspiratory pressure of the experimental group (*P* < 0.01). HR, heart rate; MAP; mean arterial pressure; MIP, maximal inspiratory pressure.

**FIGURE 6:**
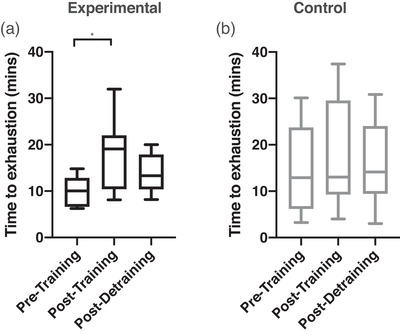
Box and whisker plot for the time to exhaustion of the loaded breathing task over various days for both groups. (a, b) Time to exhaustion at pre‐training, post‐training and post‐detraining for the experimental (a, black) and control (b, grey) group. *Significant differences (*P* < 0.05) from pre‐training.

Figure 2 shows a representative male participant in the experimental group during loaded breathing during the DETR testing day. It can be observed that both HR and MAP are attenuated compared to the average from PRE. Figure 3 shows the respiratory work at iso‐time performed during the loaded breathing trials, with no significant differences between or within groups. The rise of mean arterial pressure (percentage change and absolute change) during the loaded breathing task was attenuated during POST (−11 ± 17%, *P* = 0.003) and DETR (−9 ± 9%, *P* = 0.007) compared to PRE in the experimental group (Figure 4a, c) with no significant differences in the control group (Figure 4b, d, *P* = 0.867 and *P* = 0.914 for percentage change and absolute change, respectively). The decline in MAP was due to a decrease in both systolic and diastolic blood pressure. There was no difference in the attenuation from POST to DETR (*P* = 0.836) and the DETR levels were still lower than the PRE levels (*P* = 0.007). The attenuation of MAP is also seen in the change of absolute values. The experimental group had an attenuation of 8 ± 7 mmHg from PRE to POST (*P* = 0.027) and an attenuation of 10 ± 8 mmHg PRE to DETR (*P* = 0.01). From POST to DETR, there was no difference (−1 ± 4 mmHg, *P* = 0.868). There were no significant differences in change in MAP (percentage change and absolute) when comparing the final minute of LBT at all time points for the experimental group (*P* = 0.130 and *P* = 0.139 for percentage change and absolute change, respectively) and control group (*P* = 0.88 and *P* = 0.89 for percentage change and absolute change, respectively). There was a trend towards a significant decline in the percentage change in HR (−11 ± 14%) from PRE to POST levels in the experimental group (*P* = 0.059). No difference in the absolute change of HR (−8 ± 9 bpm, *P* = 0.147) was observed in the experimental group from PRE to POST, but HR was significantly lower on DETR compared to PRE (−11 ± 13 bpm, *P* = 0.036). There was no difference in the absolute change of HR from POST and DETR compared to PRE (−2 ± 10 bpm vs. −5 ± 14 bpm for POST and DETR, respectively, *P* = 0.088) in the control group. Figure 5a depicts the lack of relationship between the percentage change in heart rate and the change in maximal inspiratory pressure of the experimental group from PRE to POST. However, there was a significant relationship between the percentage change of mean arterial blood pressure and change in maximal inspiratory pressure of the experimental group (Figure 5b). Finally, compared to PRE, time to exhaustion in the experimental group was greater during POST (+8.1 ± 5.1 min, *P* < 0.001) but not different from DETR (+4.1 ± 5.9 min, *P* = 0.08) (Figure 6a). There were no differences in time to exhaustion in the control group (*P* = 0.10) (Figure 6b). There was no relationship between the change in MAP and the time to exhaustion (*r* ^2^ = 0.0003, *P* = 0.9668) in the experimental group.

### 3.4 Ventilation data

Ventilatory data for the control and experimental groups are presented in Tables 2 and 3, respectively. Breathing frequency, tidal volume and minute ventilation were all elevated during the loading protocol from rest, but they did not differ during the protocol or between days for either group. Similarly, end‐tidal carbon dioxide was consistent and not different between days for the latter aspects of the loading protocol. Respiratory muscle work was elevated from rest, but not different between PRE, POST and DETR for either group.

**TABLE 2:** Mean ventilation data of the control group at pre‐training (PRE), post‐training (POST) and post‐detraining (DETR) during the loaded breathing task.

	Day	F_b_ (bpm)	*V*_T_ (l)	${\dot{V}}_{E}$ (l/min)	$P_{\mathrm{ETC}O_{2}}$ (mmHg)	Work (cmH_2_O/s)	Total time (min)
Rest	PRE	9 ± 3	0.5 ± 0.2	4 ± 1	37 ± 5	−13 ± 8	—
	POST	7 ± 2	0.6 ± 0.3	4 ± 2	39 ± 5	−15 ± 5	—
	DETR	7 ± 2	0.6 ± 0.3	4 ± 1	39 ± 3	−14 ± 11	—
Minute 1	PRE	14 ± 1*	0.7 ± 0.3	10 ± 4*	38 ± 3	−100 ± 29*	—
	POST	15 ± 0.5*	0.8 ± 0.4	12 ± 6*	37 ± 2	−113 ± 26*	—
	DETR	14 ± 0.4*	0.8 ± 0.2	11 ± 3*	36 ± 4	−117 ± 28*	—
Minute 2	PRE	15 ± 0.6*	0.8 ± 0.3	11 ± 4*	38 ± 3	−104 ± 32*	—
	POST	15 ± 0.2*	0.8 ± 0.3	12 ± 4*	37 ± 4	−113 ± 27*	—
	DETR	15 ± 0.5*	0.8 ± 0.3	11 ± 4*	38 ± 4	−113 ± 25*	—
Minute 3	PRE	15 ± 0.6*	0.8 ± 0.2	12 ± 3*	38 ± 3	−107 ± 28*	—
	POST	15 ± 0.6*	0.8 ± 0.2	11 ± 4*	39 ± 2	−108 ± 26*	—
	DETR	15 ± 0.5*	0.7 ± 0.2	11 ± 3*	39 ± 4	−117 ± 23*	—
Iso time	PRE	15 ± 0.4*	0.9 ± 0.1	13 ± 2*	38 ± 3	−106 ± 23*	—
	POST	15 ± 0.4*	0.8 ± 0.2	13 ± 3*	39 ± 2	−112 ± 24*	—
	DETR	15 ± 1*	0.6 ± 0.3	9 ± 4*	41 ± 3	−114 ± 27*	—
Final minute	PRE	15 ± 0.4*	0.9 ± 0.1	13 ± 2*	38 ± 3	−106 ± 23*	14.2 ± 9
	POST	15 ± 0.5*	0.7 ± 0.2	11 ± 3*	40 ± 3	−113 ± 25*	17.4 ± 12
	DETR	15 ± 0.7*	0.6 ± 0.3	9 ± 4*	41 ± 3	−114 ± 25*	15.5 ± 9

Values are reported as mean ± SD. Work is respiratory work completed at iso‐time during the loaded breathing task (*F*_b_ × ∫*P*_mouth_ × Time).

* Significance differences from rest (*P* < 0.05).

Abbreviations: *F*_b_, breathing frequency; $P_{\mathrm{ETC}O_{2}}$ end‐tidal carbon dioxide;${\dot{V}}_{E}$, minute ventilation; V_T_, tidal volume.

**TABLE 3:** Mean ventilation data of the experimental group at pre‐training (PRE), post‐training (POST) and post‐detraining (DETR) during the loaded breathing task.

	Day	*F*_b_ (bpm)	*V*_T_ (l)	${\dot{V}}_{E}$ (l/min)	$P_{\mathrm{ETC}O_{2}}$ (mmHg)	Work (cmH_2_O/s)	Total time (mins)
Rest	PRE	8 ± 2	0.6 ± 0.2	4 ± 2	36 ± 4	−10 ± 5	—
	POST	8 ± 4	0.6 ± 0.5	4 ± 8	39 ± 3	−10 ± 2	—
	DETR	7 ± 3	0.6 ± 0.3	4 ± 4	38 ± 3	−10 ± 4	—
Minute 1	PRE	15 ± 0.6*	0.8 ± 0.6	12 ± 8*	38 ± 4	−123 ± 37*	—
	POST	15 ± 0.8*	1.1 ± 0.3	16 ± 5*	34 ± 4*	−110 ± 23*	—
	DETR	15 ± 0.7*	1.1 ± 0.3	17 ± 4*	34 ± 4*	−115 ± 35*	—
Minute 2	PRE	15 ± 0.2*	0.9 ± 0.3	14 ± 5*	38 ± 4	−124 ± 41*	—
	POST	15 ± 0.4*	1.1 ± 0.3	16 ± 5*	34 ± 4* ^†^	−109 ± 24*	—
	DETR	16 ± 1.5*	1.1 ± 0.3	17 ± 3* ^†^	35 ± 7	−117 ± 36*	—
Minute 3	PRE	15 ± 0.7*	0.9 ± 0.4	13 ± 5*	39 ± 5	−128 ± 39*	—
	POSE	15 ± 0.5*	1.1 ± 0.4	17 ± 6*	35 ± 4*	−109 ± 22*	—
	DETR	16 ± 1.8‐	1.0 ± 0.2	16 ± 3*	36 ± 4	−121 ± 38*	—
Iso time	PRE	15 ± 0.5*	0.9 ± 0.5	15 ± 7*	40 ± 6	−131 ± 43*	—
	POST	15 ± 0.8*	1.1 ± 0.3	16 ± 6*	39 ± 3^a,b,c^	−114 ± 26*	—
	DETR	15 ± 0.9*	0.9 ± 0.1	14 ± 2*	39 ± 4	−123 ± 36*	—
Final minute	PRE	15 ± 0.5*	0.9 ± 0.5	15 ± 7*	40 ± 6	−131 ± 43*	9.9 ± 3.2
	POST	16 ± 2*	1.4 ± 0.7	21 ± 10*	39 ± 5^a,b,c^	−113 ± 32*	18.0 ± 7.8^†^
	DETR	15 ± 2*	1.0 ± 0.5	14 ± 7*	40 ± 4	−125 ± 41*	13.9 ± 4.1

Values are reported as means ± SD. Work is respiratory work completed at iso‐time during the loaded breathing task (*F*_b_ × ∫*P*_mouth_ × Time).

* Significance differences from rest (*P* < 0.05).

^†^ Significance difference from pre‐training (*P* < 0.05).

Abbreviations: *F*_b_, breathing frequency; $P_{\mathrm{ETC}O_{2}}$, end‐tidal carbon dioxide; ${\dot{V}}_{E},$ minute ventilation; *V*_T_, tidal volume.

^a,b,c^Significance differences from minutes 1, minutes 2, minutes 3, respectively (*P* < 0.05).

## 4 DISCUSSION

### 4.1 Major findings

We sought to determine the strength response to training and the withdrawal of specific training, termed ‘detraining’, of respiratory muscles and an akin locomotor muscle (dorsiflexors). Additionally, we questioned if the attenuation of the respiratory muscle metaboreflex would persist after 5 weeks of detraining. The main findings in this study are twofold. First, the training‐induced attenuation of the respiratory muscle metaboreflex was lessened but persisted in the absence of IMT. We interpret this to indicate that the attenuation in respiratory muscle metaboreflex is potentially associated with changes in relative respiratory muscle strength. Second, respiratory muscle and dorsiflexor strength gains induced by significant training persisted after 5 weeks of detraining. That respiratory muscle and dorsiflexors had similar temporal responses to (de)training supports the notion that the respiratory muscles are similarly trainable to dorsiflexors, a peripheral skeletal muscle. Overall, our study provides insight into the temporal resolution of the trainability of the respiratory muscles and the impact on the cardiovascular response.

### 4.2 Respiratory muscle metaboreflex

There was an attenuation of MAP from PRE to POST by 11 ± 7% (*P* = 0.003), which is similar to Witt et al's. finding of an attenuation of 11% in MAP after 5 weeks of IMT (Witt et al., 2007). The increase of MAP during the loaded breathing task is due to increases in systolic blood pressure and diastolic blood pressure in both groups, which indicates an increase in sympathetic response. One explanation for the attenuation in MAP is that after training the MIP has increased but the resistive load remains the same, and therefore the same resistance during the loaded breathing task is now at a lower relative intensity. There is a strong inverse relationship between the changes in MAP and the changes in MIP from PRE to POST (Figure 5, *r* ^2^ = 0.7473, *P* = 0.0056) in the experimental group. This inverse relationship between the change in MAP and the change in MIP indicates that the greater increase in MIPs results in a greater attenuation of MAP during the loaded breathing task and supports that the lower relative intensity likely plays a role in the attenuation of the respiratory muscle metaboreflex, which is seen in this current longitudinal study. In agreement, a previous cross‐sectional study also found that a lower relative intensity will result in a lower MAP response during dynamic contractions in humans (Stebbins et al., 2002). Of note, we did not find a significant difference in resting MAP in the experimental group after training. The lack of change in resting MAP contrasts with others (DeLucia et al., 2018) who showed a ∼4 mmHg decrease in MAP after an approximately similar inspiratory muscle training regime in young healthy adults. The discrepancy is likely due to physiological and logistical reasons. Physiologically, the previous work demonstrated an ∼34% increase in MIP (DeLucia et al., 2018) compared to 18% increase we observed, and this greater response likely resulted in a greater effect on resting MAP. Our study was likely underpowered to detect a difference in resting MAP as our sample size (*n* = 8) was based on our primary question regarding attenuation of MAP during the metaboreflex. Indeed, the experimental group in DeLucia et al. (2018) was 50% larger and we note that our resting MAP was trending towards statistical significance (*P* = 0.08). As such, we emphasize that our lack of statistical effect of IMT on MAP is unlikely indicative of a lack of physiological effect.

Another explanation for the attenuation in MAP is that the training resulted in the improvement of muscle recruitment and changes in skeletal muscle composition. Neurological adaptations such as improved motor unit firing synchronization, faster firing frequency and decreased agonist–antagonist coactivation mean that respiratory muscles would have an improved ability to manage the same stress, resulting in lower metabolite production for a given amount of work/stress (Häkkinen et al., 1998; Kamen & Knight, 2004; Milner‐Brown & Lee, 1975). In clinical populations that experience chronically higher respiratory muscle workloads (i.e., in chronic heart failure/chronic obstructive pulmonary disease patients), over time there are changes in muscle fibre type from type II to type I, which will increase oxidative capacity (Levine et al., 1997; Tikunov et al., 1997). However, these changes are seen in patients with chronically higher respiratory muscle workload conditions over long periods of time (months to years). A change in muscle composition from type II to type I fibres is unlikely to have occurred in the present study as a previous study found that 5 weeks of strength training only resulted in a shift to type IIa from type IIx and no change in type I fibres (Plotkin et al., 2021).

The effect of training on the metaboreflex response could explain the attenuation of MAP. The diaphragm is rich in group III and group IV afferent nerve fibres and training results in a decrease in mechanically sensitive muscle afferent nerve fibre discharge (Sinoway et al., 1996). During diaphragm fatigue, there is an increase in the type IV (metabolic) afferent activity with no change in the activity of type III (mechanical) afferent nerve fibres (Hill, 2000). More importantly, trained limbs have a reduced sympathetic response compared to the untrained limb at a given pH (Sinoway et al., 1992). These adaptations could explain the attenuation in MAP seen in the experimental group. Additionally, when metabolites are above a certain threshold, they seem to have a synergistic effect on the sympathetic response (increase in HR, MAP) when mechanoreceptors are stimulated (Sinoway et al., 1993). Therefore, with changes that result in less metabolites being produced, there will be a decrease in the activity of both the mechanically (type III) and metabolically (type IV) stimulated afferents.

There was no significant change in HR at iso‐time (percentage and absolute) in both experimental and control group at POST compared to PRE. However, it was significantly lower at DETR compared to PRE (−11 ± 13 bpm, *P* = 0.038) in the experimental group. One possible explanation for the lack of difference in HR despite an attenuation in MAP is the difference in control mechanisms, with HR more influenced by central command and having efferents from both the sympathetic and parasympathetic nervous systems. Evidence for this arises from previous work using a neuromuscular blockade during static handgrip exercise (Victor et al., 1989). With the neuromuscular blockade, no force was generated even during a maximal volitional effort (Victor et al., 1989). However, the effort still resulted in an increase in HR, similar to that of performing 30% of maximal voluntary contraction, indicating that the rise in HR is in part due to central command (Victor et al., 1989). On the other hand, MAP increased minimally during the contraction with neuromuscular blockade and was significantly lower compared to the 30% maximal voluntary contraction. Since HR values in our study were compared at iso‐time, where the work completed was similar, the contribution of central command to the response of HR should also be similar. The small difference in the attenuation of HR response is likely due to the neural adaptations (i.e., improved recruitment patterns) and contributed to minimal differences in HR (−11 ± 14%).

There was an increase in time to exhaustion in the experimental group after 5 weeks of training (Figure 6), which was expected given the increase in strength (Sawyer et al., 2014). Time to exhaustion was not different between POST and DETR after the 5 weeks of no training, but it was trending towards returning to PRE levels. However, there was no relationship between the change in MAP and the change in time to exhaustion, indicating that the increase of time to exhaustion is not exclusively due to changes in the strength or respiratory metaboreflex. The lack of a relationship between MAP and time to exhaustion may suggest that other factors play a role in this increase in time to exhaustion during the loaded breathing task from PRE (first time) to POST (second time) in the experimental group. One potential factor is the familiarization effect. A single familiarization session of a given exercise task will induce a learning effect that will enhance performance in subsequent sessions of that exercise task (Corbett et al., 2009; Noreen et al., 2010). However, in the current study there was no difference in time to exhaustion throughout the three testing days (PRE, POST and DETR) in the control group but there was an improvement seen in the experimental group, suggesting that the effect of familiarization is minimal for this task (Figure 6). Another explanation for the improvement in time to exhaustion is that the strength training improved anaerobic capacity, which could also lead to an improvement in time to exhaustion (Sawyer et al., 2014). However, the improvement in time to exhaustion is unlikely a result of the improvement in anaerobic capacity because anaerobic capacity improvements were seen after 8 weeks of training and the changes of muscle fibre type occurred after 6 weeks, while our training protocol was only 5 weeks (Plotkin et al., 2021; Sawyer et al., 2014). Additionally, the strength of the MIPs remained elevated after DETR (Figure 1a). However, the time to exhaustion was already trending towards back to PRE levels (Figure 6), supporting that strength unlikely played a role in the time to exhaustion.

### 4.3 Strength response

Based on the changes in MIP and dorsiflexor strength, our data indicate that the respiratory muscles and dorsiflexors respond temporally similarly to training and detraining. That is, both showed an increase in force/pressure from the 5 weeks of training and no significant changes to 5 weeks without training (Figure 1 and Table 1). The increase of MIP from PRE to POST (+18%) was similar to that seen by others who employed a similar training regime involving two sets of 30 repetitions for 5 weeks at 50% of MIP (+16% for Ramsook et al. (2017) and +17% for Witt et al. (2007)). While our study and others demonstrated a robust increase in MIP, this does not appear to be at a ‘plateau point’ of strength gains as a similar IMT protocol over 9 weeks resulted in a 41 ± 1% increase in MIP (Romer et al., 2002).

The increase in strength seen in both respiratory muscles and TA is less likely due to hypertrophy as minimal hypertrophy occurs at the beginning stages of resistance training (McGlory et al., 2017). However, as the participant completes more of the training protocol, more of the strength gains can be attributed to hypertrophy (Moritani & DeVries, 1979). Since the training protocol in this study was only 5 weeks, the increase in strength is more likely attributable to neurological adaptations, occurring during earlier stages of training (Pearcey et al., 2021). Some of the neurological adaptation would include improved motor unit firing synchronization, faster firing frequency, and decreased agonist–antagonist coactivation (Häkkinen et al., 1998; Kamen & Knight, 2004; Milner‐Brown & Lee, 1975). Any one of these neurological adaptations will improve muscle strength, but it is likely a combination of all these adaptations. These neural adaptations can vary between skeletal muscles based on differences in daily usage and activity levels, resulting in being more or less trained (Sale, 1988). Specifically, a muscle that is used regularly is likely to be more neurologically adapted, and therefore when introducing a training stimulus, it is likely to have an overall smaller increase in strength. In the present study, 5 weeks of training resulted in a strength increase of 34 ± 15% vs. 18 ± 8% for TA and respiratory muscles, respectively. Even though the TA and respiratory muscles have comparable traits (both are skeletal muscles, muscle fibre types, daily utilization in healthy individuals) their response was different. The greater gain of strength in the TA compared to respiratory muscles is likely because the TA is not continuously active (i.e., when sitting, sleeping, etc.). Due to the constant state of respiratory muscle activation, the diaphragm may be more neurologically and mechanically trained than the TA, and therefore it is not surprising that the TA had a greater strength gain (Ahtiainen et al., 2003).

The percentage change (gain) in MIP did not have a relationship with the starting MIP (*r* ^2^ = 0.049, *P* = 0.5992). The gain of MIP varied from individuals and one possibility for this difference is that the starting MIPs are absolute values and not relative values. Therefore, even though a participant may have a higher starting MIP, it does not necessarily indicate that their respiratory muscles are more trained as it does not take into consideration other factors (i.e., height, size).

There was no difference in MIPs from POST to DETR, indicating the respiratory muscle strength remained elevated after 5 weeks of no IMT, and this finding is supported by the finding of another study where MIPs remained elevated after 9 weeks of detraining (Romer et al., 2002). An important consideration is that the increase in MIP occurred without changes in MEP in either group (Figure 1). The lack of change in MEP confirms the specificity of our training stimulus and indicates that our increase in MIP was unlikely due to other factors such as familiarization or other training.

### 4.4 Methodological considerations

Our study has methodological considerations that warrant discussion. First, while the dorsiflexors share many functional and anatomical features of the respiratory muscles, there are important differences. Chiefly, the respiratory muscles are more active than dorsiflexors because they require continuous activation to sustain alveolar ventilation. Second, photoelectric plethysmography to measure beat‐by‐beat blood pressure during a loading breathing task can result in artefacts due to the impact large inspiratory pressure generation has on peripheral blood. Normally, the photoelectric plethysmography performs a calibration after every 70 beats, but our loaded breathing task resulted in additional calibrations during the data collection. Photoelectric plethysmography is also sensitive to contraction/movement of the hand/fingers. To minimize this, our participants kept their hand still and relaxed while being supported by a table.

Third, to make appropriate comparisons of the cardiovascular impact of loaded breathing, the exercise task and work performed need to be similar. Indeed, the cumulative work at iso‐time (Figure 3) and average work (Tables 2 and 3) at each time point was not different between PRE, POST and DETR in either group. As such, our physiological response (i.e., changes in HR and MAP) were being compared when the participants were experiencing the same magnitude of the stressor (i.e., respiratory muscle work). Fourth, to ensure our training was localized to the inspiratory respiratory muscles, we had the participants perform MEPs during each visit. As the MEPs were not different between all three visits for both groups, we are confident our training was isolated to the inspiratory muscles and our observed effect induced by the intervention was unlikely due to learning/familiarization. Fifth, we did not directly measure sympathetic activity during loading trials. However, it is well established that changes in HR and MAP are associated with changes in sympathetic activity (Gordan et al., 2015) and acquiring a sympathetic recording for three separate experimental trials was not currently feasible. Sixth, we elected to test the metaboreflex after 5 weeks without IMT (DETR), which was the same duration as training. In the 5 weeks without IMT, there was minimal change in MIP and thus the inspiratory muscles were not detrained per se, but rather we removed the training stimulus. Our goal was to evaluate the change in metaboreflex after the equivalent time without IMT, and thus it was not requisite to have a decrease in MIP. However, subsequent studies should investigate longer detraining times where inspiratory muscle force is significantly reduced and evaluate any potential impact on the metaboreflex. Lastly, there are notable sex differences in the respiratory muscle metaboreflex with females showing a blunted MAP response (Welch et al., 2018). To address this, we ensured there were approximately similar females in both groups, and employed a longitudinal study design where females taking monophasic birth control were included.

### 4.5 Conclusion

In conclusion, 5 weeks of inspiratory muscle and dorsiflexor training resulted in similar temporal strength responses from both muscle groups as strength increased in both muscle groups and remained elevated after 5 weeks of detraining. In addition, 5 weeks of IMT also attenuates the respiratory metaboreflex, an effect that persisted after 5 weeks of detraining, which may have other benefits (i.e., reduced competition of blood flow with other muscles during exercise). Our findings suggest that the benefits of IMT persist after 5 weeks of detraining.

## AUTHOR CONTRIBUTIONS

Experimental design: J.S.C., M.D.A., R.L.H., P.B.D. Data acquisition: J.S.C., L.M.M., C.J.D., S.A.A., B.P.T., P.B.D. Data analysis: J.S.C., L.M.M., C.J.D., S.A.A., B.P.T., P.B.D. All authors, interpreted, drafted and approved the final version. All authors agree to be accountable for all aspects of the work in ensuring that questions related to the accuracy or integrity of any part of the work are appropriately investigated and resolved. All persons designated as authors qualify for authorship, and all those who qualify for authorship are listed.

## CONFLICT OF INTEREST

None.

## Supporting information

Statistical Summary Document

## Data Availability

The data that support the findings of this study are available from the corresponding author upon reasonable request.
